# The Mediterranean Lifestyle to Contrast Low-Grade Inflammation Behavior in Cancer

**DOI:** 10.3390/nu15071667

**Published:** 2023-03-29

**Authors:** Rosa Divella, Graziella Marino, Stefania Infusino, Laura Lanotte, Gaia Gadaleta-Caldarola, Gennaro Gadaleta-Caldarola

**Affiliations:** 1 Unità Operativa Complessa di Oncologia, Ospedale “Mons. A. R. Dimiccoli”, Asl BT, Viale Ippocrate 15, 76121 Barletta, Italy; 2 Department of Breast Unit—Centro d Riferimento Oncologico della Basilicata, IRCCS-CROB, Via Padre Pio 1, 85028 Rionero in Vulture, Italy; 3 Unità Operativa Complessa di Oncologia, Ospedale “SS Annunziata”, Via Felice Migliori 1, 87100 Cosenza, Italy; 4 Scienze e Tecnologie Alimentari, Università di Parma, Via Delle Scienze 59/A, 43124 Parma, Italy

**Keywords:** lifestyle, cancer, nutrition, physical activity

## Abstract

A healthy diet and an active lifestyle are both effective ways to prevent, manage, and treat many diseases, including cancer. A healthy, well-balanced diet not only ensures that the body gets the right amount of nutrients to meet its needs, but it also lets the body get substances that protect against and/or prevent certain diseases. It is now clear that obesity is linked to long-term diseases such as heart disease, diabetes, and cancer. The main reasons for people being overweight or obese are having bad eating habits and not moving around enough. Maintaining weight in the normal range may be one of the best things to avoid cancer. It has been scientifically proven that those who perform regular physical activity are less likely to develop cancer than those who lead a sedentary lifestyle. Moving regularly not only helps to maintain a normal body weight, avoiding the effects that favor tumor growth in overweight subjects, but also makes the immune system more resistant by counteracting the growth of tumor cells. Physical activity also helps prevent cardiovascular and metabolic diseases. In this review, it is highlighted that the association between the Mediterranean diet and physical activity triggers biological mechanisms capable of counteracting the low-grade chronic inflammation found in patients with cancer. This assumes that healthy lifestyles associated with cancer therapies can improve the expectations and quality of life of cancer patients.

## 1. Introduction

Approximately 30–35% of all malignancies are attributed to so-called lifestyle factors, which include an imbalanced diet, being overweight or obese, abusing alcohol, and inactivity [1]. Obesity, defined as abnormal excess accumulation of fat in adipose tissue, causes chronic low-grade inflammation. It is associated with a high risk of developing the four main types of non-communicable diseases (NCDs), namely, type 2 diabetes, metabolic syndrome, cardiovascular disease, and several types of cancer [2,3]. In tumors of the breast, colon, liver, and prostate, obesity is generally a predictor of poor clinical outcomes. Consequently, diet, viewed as the totality of eating habits, is a significant environmental component capable of regulating neoplastic risk either as a protective factor or as a risk factor, depending on the quality, amount, and frequency of intake of the foods that comprise it [4]. The relationship between nutrition and the immune system is crucial to both sustaining health and causing illness. When the immune system is hyperactive as a result of an unhealthy lifestyle and an imbalanced diet, a low-grade chronic inflammatory state is induced, which increases the risk of developing chronic degenerative illnesses and cancers [5,6]. Nutrition has a dual nature because it can be either a weapon of prevention and therapy or a cause of pathological events if it is primarily composed of pro-inflammatory foods consumed on a regular basis. Diets, such as the traditional Mediterranean diet (MD), and regular physical exercise are effective methods for preventing overweight, obesity, and NCDs, as well as reducing visceral fat and, as a result, the generation of pro-inflammatory cytokines and hormonal imbalances [7,8]. Moreover, the UNESCO in 2010 officially defined the MD as a cultural heritage of humanity, exalting conviviality, sensory stimulation, socialization, biodiversity, and seasonality, aspects that can reinforce the MD’s beneficial effects on wellbeing, quality of life, and healthy physical activity. Furthermore, a balanced diet is now regarded as a very effective tool for the prevention and reduction of mortality and the recurrence of oncological illnesses [9,10]. These intrinsic characteristics of the Mediterranean diet allow us to place it in a really healthy lifestyle, which is reflected in the culinary and working traditions (fishing and sheep farming) of the Mediterranean people, which require intense physical effort (far from a sedentary life) and, therefore, high energy consumption. 

Physical exercise benefits cancer patients, including those receiving oncological treatments such as chemotherapy, hormone therapy, radiation, and surgery, as well as those in the rehabilitation phase and long-term survivors [11]. Physical exercise is now shown to be safe for cancer patients and must be included in treatment regimens, even though it must be tailored to the subject being treated, the kind of pathology, comorbidities, physical conditions, and effort tolerance [9]. Physical exercise, when combined with a balanced diet and a proper lifestyle (no smoking or drinking), leads to a prolongation of the expected lifespan and a decrease in the risk of chronic degenerative illnesses and tumors, which are statistically common in the adult population [12]. This review focuses attention on the importance of adopting a healthy lifestyle defined as a Mediterranean lifestyle where nutrition and physical activity become the two fundamental building blocks for achieving a good state of health and quality of life in cancer patients.

## 2. Physical Activity and Inflammation

Physical activity, especially when performed moderately and on a regular basis, has an anti-inflammatory effect and lowers inflammation markers [13]. Muscle contraction causes the release of proteins called myokines, some of which have local and systemic endocrine effects and are involved in lipid, glucose, and bone metabolism, and hence contribute to the anti-inflammatory effects of exercise [14,15]. In addition to aiding in weight regulation; preventing diabetes type 2, insulin resistance, and metabolic syndrome; and enhancing cognitive abilities and slowing the progression of neurodegenerative diseases such as Parkinson’s and Alzheimer’s, it plays an essential role in the body’s metabolic processes [16,17]. Additionally, it may halt the development of cancer, making it useful both for primary cancer prevention and to cancer survivors [18]. Recent research indicates that even moderate exercise on a treadmill for 20 to 30 min can stimulate the immune system and produce an anti-inflammatory response, resulting in a decrease in TNF-α (tumor necrosis factor-alpha) and a beneficial effect on low-grade chronic inflammation [19].

## 3. From Sedentary Lifestyle to Chronic Inflammation Status

**Figure 1:**
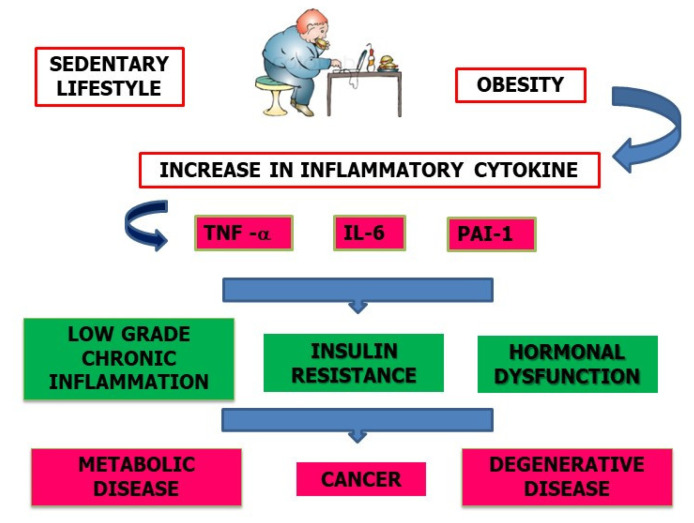
Schematic representation of the consequences due to a sedentary lifestyle.

Sedentary living is defined by excessive inactivity throughout the day and frequent sitting in front of the TV, computer, or video games; at work; and in the car [20]. Energy is expended more readily when standing due to the contraction of muscles (especially those of the lower extremities), whereas sitting has been linked to detrimental effects on lipid metabolism. Further, eating while seated is linked to weight gain, which in turn raises the risk of developing metabolic syndrome and obesity [21]. Under these conditions, the visceral adipose tissue is altered, resulting in changes in the production of steroid hormones and adipokines, metabolic disorders, and chronic subclinical inflammation. Low-grade chronic inflammation caused by elevated levels of tumor necrosis factor alpha (TNF-a), interleukin 6 (IL-6), leptin, and other inflammatory adipokines, including plasminogen activator inhibitor 1 (PAI-1), is a risk factor for the development of malignant neoplasms in people who are overweight or obese, Figure 1 [22,23,24]. In addition, this state of inflammation leads to hyperinsulinemia, which in turn promotes insulin resistance with the increased production of IGF-1 (insulin-like growth Factor-1) and hormone imbalances, including an increase in estrogens as a result of androgen conversion at the adipose tissue level, which in turn stimulates the proliferation of mammary gland and endometrial epithelial cells [25,26]. The chance of developing cancer due to these causes is elevated. The detection of C-reactive protein (PCR) in the blood has prognostic significance since it serves as an essential indication of the metabolic and inflammatory state caused by the liver in response to IL-6 emitted by adipocytes [27]. To live a healthy and long life free from diabetes, degenerative diseases, heart disease, and cancer, it is possible to make positive changes to one’s behavior (mental, physical, and nutritional) at any time.

## 4. Physical Activity and Cancer

Physical exercise, when properly performed in moderation and in conjunction with an anti-inflammatory diet such as the traditional Mediterranean diet, also has an essential role in preventing and fighting cancer, as has been shown by various epidemiological, observational, and meta-analysis studies [28,29]. Consistent exercise alters several biochemical markers associated with cancer cell metabolism [30]. Modulatory effects on hormones, growth factors, and cytokines are of special interest as potential pathways by which physical exercise prevents and fights cancer [31]. One of exercise’s primary effects is a decrease in plasma concentrations of the hormone estrogen. Inducing cell proliferation and progression, blocking apoptosis, and promoting angiogenesis are all ways in which estrogens promote breast carcinogenesis [32]. Several studies have revealed that menopausal women who engage in regular exercise have lower levels of free estrogen in their blood [33,34,35]. Exercise has several effects on endogenous estrogen, including weight loss, changes in serum adipokine levels, and a lower circulating insulin concentration [36,37]. The final pathway causes a rise in the hormone sex-hormone-binding globulin (SHBG), a protein that binds to estrogens and decreases their bioavailability. It is postulated that fat mass loss mediates the relationship between exercise and estrogen reduction through increased SHBG [38,39,40]. Given that fat cells produce most of the body’s endogenous estrogen after menopause, a connection between the two seems reasonable [41]. Increased circulating amounts of estrogen and/or insulin and IGF-1 may be one mechanism by which excess body fat promotes cancer development and progression in postmenopausal women (insulin-like growth factor) [42,43]. The expression of IGF-1 is greater in ER-positive tumors than in ER-negative tumors because both insulin and IGF-1 increase aromatase enzyme activity in adipose tissue, which boosts local estrogen synthesis [44,45]. As a result, an increase in adiposity is responsible for the elevated amounts of circulating estrogen seen in obese women. 

Physical exercise may be used to prevent the side effects of chemotherapy and radiation, such as weariness and nausea, during treatment times [46,47]. Exercises carried out gradually and consistently may enhance certain benefits related to the tumor’s pathology and the treatments given, lower the risk of cardiovascular disease, and prevent the reappearance of neoplastic disease [48,49]. Aerobic exercise enhances cardiovascular and respiratory health as well as strength, endurance, and musculoskeletal health in cancer patients [50,51]. Exercise prevents weight gain, diminished muscular strength, and cardiotoxicity in individuals on androgen deprivation for prostate cancer [52]. Rehabilitative exercise may help with secondary lymphedema and limb mobility in women who have had axillary dissection and a mastectomy for breast cancer [53]. In fact, when more muscle groups are engaged, lymph outflow improves. Adipose and muscle tissue may undergo epigenetic changes as a result of physical exercise, which can be shown as an increase or reduction in DNA methylation [54,55]. The most interest is being shown in research on the methylation of certain genes. For instance, Nakajima’s research demonstrates that alternating aerobic exercise (three minutes of brisk walking followed by three minutes of running) affects the methylation of the ASC gene, which regulates the cytokines IL-1beta and IL-18, important for the inflammatory response [56]. The p53 tumor suppressor gene, which is suppressed in many malignancies and causes histone alterations as well as differences in the expression of microRNAs that govern protein synthesis and the regeneration of muscle mass and fiber type, is also more likely to be expressed when people are physically active [57,58,59]. It is also crucial to schedule physical activity each week. It has been shown that individuals with colon cancer who engage in at least six hours of physical activity each week have a much higher survival rate than those who engage in less than one hour of exercise each week [60,61]. A 50,000-woman study from the Albert Einstein College of Medicine in New York indicated that individuals who maintained a lifestyle in accordance with the American Cancer Society’s guidelines had a 31% lower chance of acquiring breast cancer [62]. The combined use of physical exercise and cancer treatments such as chemotherapy has positive results [63,64]. Exercise helps cancer patients even four years after diagnosis, according to May et al. In participants with a 4-year follow up, it was noted that adverse effects, such as weariness, decreased [65]. Physical exercise has a number of beneficial biological effects, some of which have been hypothesized to explain the preventative activity, including the decrease of insulin resistance, chronic inflammation, and immune system dysfunction; the reduction of fat mass in obese individuals; and the blood levels of hormones including insulin, estrogen, and growth factors [66,67,68].

## 5. Diet and Chronic Inflammation

Chronic low-grade inflammation is at the root of chronic-degenerative illnesses in the elderly and malignancies. Sedentarism, extra food calories, an improper diet, and bad food all lead to chronic inflammation [69,70,71]. Fat accumulation, particularly visceral obesity, controls the production of pro-inflammatory cytokines, which encourage the establishment of an inflammatory environment, as well as the synthesis of hormones, and the conversion of androgens into estrogen in adipose tissue, which contribute to tumor development in the endometrium, breast, and colon [72,73,74]. Additionally, diets high in processed foods and “junk food” change the gut bacterial flora, causing dysbiosis, which worsens and perpetuates the inflammatory state [75,76]. According to recent research by Zhang et al., a poor diet seems to be a major risk factor for malignant tumors. Colorectal cancer had the largest number of diagnoses and the highest percentage connected to diet (38.3%) [77]. Relevant variables were a lack of whole grains and a high intake of processed meats. Cancer was most common in males aged 45 to 64. The study’s American authors validate the statistics given by the American Association for Cancer Research, which states that food is responsible for 5% of cancers [78]. According to the latest research, a diet rich in whole grains, vegetables, fruit, fish oil, extra virgin olive oil, prebiotic fibers, spices, and a moderate intake of red wine, such as the traditional Mediterranean diet, also with the addition of spices, may modify and positively impact the immune functions with a protective and preventative activity and can maintain the microbiota in eubiosis, safeguarding the intestinal barrier [79,80]. As a result, germs and endotoxins that might lead to persistent low-grade inflammation are kept from spreading throughout the body.

## 6. The Mediterranean Diet (MD)

Thanks to its unusual preventative effects, the MD has attracted the attention of scientists throughout the world. The Mediterranean diet constitutes a food model that characterizes not only a lifestyle but also a culture and has been indicated as a hub for improving health, quality of life, and life span. Numerous studies have highlighted the positive correlation between MD and longevity; individuals who adhere to a nutritional style such as this have a longer life expectancy [81,82]. Moreover, the Mediterranean diet prevents many metabolic, cardiovascular, and neurodegenerative diseases; insulin resistance; and different types of cancer [83,84]. Today, cancer represents the second leading cause of death in the world, immediately after cardiovascular diseases, but its onset curve has lowered in parallel with other chronic degenerative diseases, such as diabetes and obesity. In this context, diet plays a very important role. Epidemiological and clinical studies support the association between nutrition and the development or progression of different cancer malignancies such as colon, breast, prostate, and other cancers, defining these tumors as diet-associated cancers [85,86]. If followed correctly, it may help people avoid the chronic inflammation that results from being overweight, developing metabolic syndrome, and gaining excess weight due to a poor diet. In general, the Mediterranean diet is full of healthy nutrients, including antioxidants, anti-inflammatories, and insulin sensitizers [87,88]. Within several months of its implementation, it has been linked to less inflammation and has been shown to reduce inflammatory cytokines (IL-6 and TNF-alpha) and the C-reactive protein and to enhance anti-inflammatory IL-10 [89,90]. In addition to lowering insulin levels and correcting hepatic steatosis, the MD has been shown to reverse the metabolic syndrome in a significant number of patients. It is a healthy routine that has been shown to reduce cancer risk [91,92]. This is because eating these foods together helps prevent DNA damage, cell proliferation, and the survival of cancer cells by lowering oxidative and inflammatory processes inside the body [93]. Furthermore, in the DIMENU study (Dieta Mediterranea and Nuoto), according to the data reported in the literature, serum from adolescents with high adherence to the Mediterranean diet reduced inflammation in human macrophage in vitro models, suggesting that it could have a positive impact on the prevention of chronic diseases, including cancers in adulthood [94].

## 7. Role of an Anti-Inflammatory and Calorie-Restricted Diet in Cancer Prevention

**Figure 2:**
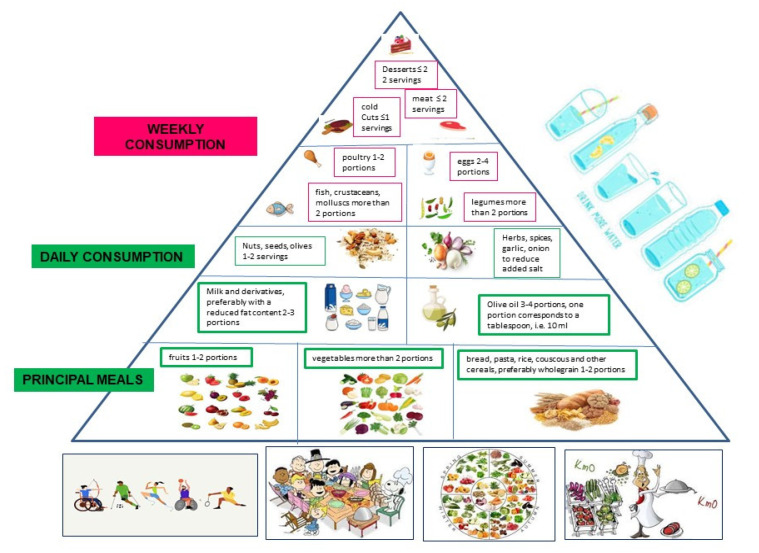
The typical Mediterranean diet and lifestyle raccomandations to follow: Mediterranean Lifestyle.

The typical Mediterranean diet can help avoid cancer. To achieve this objective, it is recommended to follow the diet following the suggestions below and illustrated in the model of the Mediterranean diet pyramid illustrated in Figure 2.

### The Typical Mediterranean Diet Recommendations to Follow

(1) Consume whole grains every day, three portions a day of vegetables, two portions a day of fruit, and legumes 3–4 times a week.
(2) Limit the consumption of red meat, avoid processed meats such as preserved meats, cold cuts, and frankfurters.
(3) Stay away from items high on the glycemic index, such as white bread, white rice, and simple sweets. Dinner carbs are acidifying and may raise blood sugar and insulin levels, so it is best to avoid them.
(4) Fruit is best eaten on an empty stomach, either first thing in the morning or late in the afternoon and never toward the conclusion of a meal, especially one that is high in carbs. Do not eat fruit salad since it contains ingredients with wildly varying pH levels, many of which might lead to stomach and bowel issues.
(5) Foods high in acidity and polyamines should be avoided (vegetables of the nightshade family, oranges, tangerines, mandarin oranges).
(6) Stay away from packaged foods with hydrogenated polyunsaturated fatty acids (trans), which cause high cholesterol and inflammation throughout the body, which damages cells. Trans fats can be found in bakery products such as industrial bread, cookies, and pastries, as well as in ready-to-eat meals and French fries.
(7) Food supplements should not be taken unless they have been properly tested in clinical trials.
(8) Observe a moderate calorie restriction while making sure that all essential nutrients are in the diet. This is to make sure that the nutritional status is not affected (for example 1–2 days a week).
(9) Weight should be managed in a healthy way; one should “stay trim” and monitor one’s waist circumference (waist size), which should be no more than 80–88 cm in women and 94–102 cm in males.
(10) Even more so as you get older, it is important to get your body moving every day; try going for a brisk 30 min walk, walking 10,000 steps, or spending an hour at the gym.

Numerous studies suggest that calorie restriction decreases plasma insulin, glucose levels, sex hormones, and inflammatory cytokines; increases detoxifying enzymes; decreases oxidative stress; and increases adiponectin, an anti-inflammatory protein produced by adipocytes [95,96,97]. When the diet contains all the required nutrients, 25–30% less calories in the diet lengthens animal life and minimizes the occurrence of cancers [98]. In fact, it has been shown that calorie restriction stimulates the AMPK (AMP-activated protein kinase) protein, which decreases the production of mTOR (mammalian target of rapamycin), one of the major oncogenes and regulators of cell growth and proliferation [99,100]. When caloric restriction is accompanied by a drop in animal protein, plasma levels of IGF-1 decline, which, in conjunction with insulin, activates the PI3K–Akt–mTORC signaling pathway [101]. As an alternative to mitochondrial respiration, the latter may stimulate cell growth and aerobic glycolysis, which constitutes the primary energy source of cancer cells. Aerobic glycolysis generates lactic acid and acidifies the milieu in which tumors thrive; the acidity increases tumor dissemination by boosting angiogenesis through VEGF (vascular endothelial growth factor) [102]. It has been shown that even brief periods of fasting (a couple of days a week) lower blood sugar, insulin, and IGF-1 levels and enhance the efficacy of oncological therapies [103,104]. By blocking mTOR, calorie restriction is in fact able to limit cancer cells’ DNA repair capabilities. In the days before chemotherapy, calorie restriction might be prescribed to cancer patients. In conclusion, the key points of physical exercise and a healthy Mediterranean diet are given below:

- Modulates insulin levels, reduces insulin resistance and insulin-like growth factor (IGF-1).
- Fights against weight gain and obesity.
- Decreases the production of inflammatory adipokines and leptin (mitogenic factor) while increasing the production of adiponectin (pro-apoptotic factor) by adipocytes with anti-inflammatory and antitumor action.
- Reduces plasma levels of estrogens, which are involved in the growth of breast and endometrial cancer cells.
- Contributes to the anti-inflammatory action with the muscular release of anti-inflammatory myokines whose protective effect in many tumors has been demonstrated.
- Increases intestinal motility, thereby reducing the contact time of the intestinal mucosa with carcinogenic compounds.
- Modifies the composition and metabolic profile of the microbiota, exerting a protective action in inflammatory bowel diseases.

## 8. Conclusions

The American Institute for Cancer Research and the World Cancer Research Fund estimate that following appropriate dietary guidelines, engaging in consistent daily physical exercise, and maintaining a healthy weight would prevent 30–40% of all cancers. Additionally, the same treatment might lower mortality from pancreatic, prostate, colorectal, breast, and other cancers by up to 50%. When it concerns prostate cancer, dietary and lifestyle modifications not only have the obvious effect of preventing the neoplasm but also of regulating its behavior in the development stages, either by slowing down the evolution of the tumor when it is already present or by preventing its return after a drastic surgery [105,106]. From a physiological point of view, physical activity improves insulin sensitivity, lowers visceral body fat by bringing the adipocytes back into a state of balance in the production of adipokines favoring anti-inflammatory ones, and lowers estrogen levels, as well as inflammation markers such as CRP, which together facilitate cancer formation. Physical exercise may lower the probability of developing cancer, Friedenreich et al. reports a 25% reduction in the risk of breast cancer, cancerous cell proliferation, and the side effects of adjuvant therapy [107]. High-intensity workouts carried out for a short period of time have also been shown to benefit patients with different forms of cancer [108]. In conclusion, it seems adequate to engage in 150 to 300 min per week of aerobic physical activity of moderate intensity, or 75 to 150 min of vigorous and intensive activity, in order to gain a health advantage. The combination of a balanced diet with an active lifestyle is an effective strategy for the prevention, management, and treatment of several illnesses [109]. A healthy and balanced diet not only ensures an appropriate supply of nutrients capable of meeting the body’s demands but also permits the incorporation of chemicals that play a protective and/or preventative function against specific pathological disorders. A diet abundant in fruit, vegetables, legumes, fatty fish with a high content of omega-3 fatty acids, and extra virgin olive oil; a balanced ratio between omega-6 and omega-3 fatty acids; the daily intake of foods containing physiologically active herbal components; and the use of fish oil supplements all show preventative activities [110,111]. 

Dietary and lifestyle interventions may result in functional alterations of the endocrine system, a decrease in inflammatory events, and regulation of genetic transcription [112,113,114]. Considering that cancer is a disease caused by genetic abnormalities that come from damage to cellular DNA, this component is crucial. It has been established that various lines of intervention have beneficial effects not only on the cardiovascular system but also on tumor pathology, slowing the evolution of tumors. The main focuses of cancer supportive care are on enhancing survival, motivating patients to take action, and lessening the burden of treatment-related side effects. Cancer treatment plans should always incorporate complementary measures, such as nutrition and exercise. International guidelines and scientific evidence show that a well-planned diet and exercise program may reduce or eliminate many of the negative consequences of both the ailment and its treatment. Despite a growing amount of evidence, sports and physical therapy and nutritional therapy are seldom included into standard cancer treatment plans. Interprofessional communication is crucial for incorporating dietary and physical interventions, as well as sports therapy, into cancer care.

## Data Availability

The study did not report any data.
